# Supplementary material for: A Systematic Compilation of Human SH3 Domains: A Versatile Superfamily in Cellular Signaling
Source: Cells. 2023 Aug 12;12(16):2054. doi: 10.3390/cells12162054 (PMC10453029; doi:10.3390/cells12162054)
Supplement: Supplementary file 1 [file cells-12-02054-s001.zip › cells-2511053-supplementary.pdf]

Supplementary Information

A Systematic Compilation of Human SH3 Domains: A Versatile Superfamily in Cellular Signaling

Mehrnaz Mehrabipour, Neda S. Kazemein Jasemi, Radovan Dvorsky and Mohammad R. Ahmadian
Institute of Biochemistry and Molecular Biology II, Medical Faculty and University Hospital Düsseldorf, Heinrich Heine University Düsseldorf, 40225 Düsseldorf, Germany

Phylogenetic analysis

To identify proteins containing SH3 domains, we employed an advanced search technique with a combination of sequence similarity identification. The initial step involved identifying SH3 domain-containing proteins (SH3DCPs) which was accomplished by conducting a comprehensive search using available UniProt protein database. From the total pool of 394,887 SH3DCPs identified across various organisms, we focused on a subset of 1,132 proteins that had undergone review. In the case of human SH3DCPs, 237 out of 770 proteins were subjected to further analysis and characterization. In the next step, we employed a sequence comparison approach using the ClustalW algorithm to align and compare the input sequences to accurately identify the regions annotated as SH3 domains within the SH3DCPs. This analysis led to the discovery of 298 SH3 domains embedded in 221 human SH3DCPs.
To address the classification and categorization of SH3 domain-containing proteins (SH3DCPs) considering their diverse domain compositions, we employed an approach based on similarities in domain compositions between these proteins. For this purpose, we focused on the collected 221 human SH3DCPs retrieved in the previous steps. To retrieve the primary sequences of these proteins, we accessed the UniProt database and extracted the necessary data for analysis. The primary sequences were then analyzed to identify the occurrence of protein domains within each SH3DCP. This analysis aimed to capture the domain composition diversity across the entire collection. To evaluate the mutual similarities in domain composition between all protein pairs in the collection, a matrix was generated. This matrix represented the similarities and dissimilarities in domain compositions among the SH3DCPs. Next, the resulting matrix was subjected to phylogenetic analysis using the MEGA software (version 7.0). The phylogenetic analysis aimed to uncover the evolutionary relationship within the human SH3DCP superfamily based on their domain compositions. By analyzing the generated phylogenetic tree, we were able to classify the human SH3DCP superfamily into thirteen distinct SH3DCP families (Figure 3). This classification provided insights into the evolutionary relationships and allowed for a more comprehensive understanding of the diversification and organization of SH3DCPs. This approach, utilizing sequence analysis, domain composition comparison, and phylogenetic analysis, facilitated a meaningful classification of SH3DCPs based on their heterogeneous domain compositions. To support this analysis, we created a supplementary Table (Table S1) to document the specific SH3 domains found within each human SH3DCP, providing further insights into the functionality and distribution of SH3 domains in the human proteome.

Domain organization

In order to analyze the domain composition of SH3DCPs, we first collected sequences of all human proteins from UniProt that contain at least one SH3 domain. All collected proteins were then scanned for protein domains using the utility hmmscan from the HMMER package against the protein domains profile database obtained from Pfam. Outputs from hmmscan were parsed with Python programming language utilizing the Biopython library and domain composition for each protein in the collection was retrieved. Similarities and/or differences in domain composition for each pair of proteins were then calculated with Python script in the form distance matrix which was then used in MEGA software to generate a phylogenetic tree by UPGMA method. The phylogenetic tree from the previous step was finally used for clustering/grouping of SH3DCp while a graphical representation of domain composition for each protein, generated using Python image libraries OpenCV and Pillow, was added to it for better visualization.

Table S1. Phylogenetic classification of the human SH3DCP superfamily into thirteen families.

Fam	Entry name (no. of SH3 dom)	Aliases, interactions & functions[a]	Uniprot ID[b]	References
1: SH2 and/or KinYST domains	TXK (1)	PTK4; BTKL; RKL; regulates the development and differentiation of conventional T-cells	P42681	-
	YES (1)	HST441; regulates cell growth, adhesion, cytoskeleton remodeling, and differentiation	P07947	-
	SRC (1)	THC6; ASV; participates in transcription, immunity, adhesion, apoptosis, migration	P12931	1-6
	MATK (1)	CHK; CTK, HYL; LSK; has an inhibitory role in the control of T-cell proliferation	P42679	-
	LCK (1)	LSK; YT16; targets RUNX3, PYK2, MAPT, RHOH and TYROBP in T-cell regulation	P06239	7
	FYN (1)	SLK; SYN; regulates cell growth and survival, adhesion, motility, & axon guidance	P06241	8-11
	FGR (1)	SRC2; regulates immune responses via AKT1, ABL1, CBL, CTTN, FAK1, PYK2 & VAV2	P09769	-
	BLK (1)	MODY11; p55; B-cell receptor signaling & development	P51451	-
	CSK (1)	CYL; regulates cell growth, migration & immune response	P41240	-
	FRK (1)	PTK5; RAK; GTL; stabilizes PTEN & negatively regulates cell proliferation	P42685	12
	HCK (1)	p59; targets ADAM15, BCR, ELMO1, GAB1", RAPGEF1, STAT5B, TP73, VAV1 & WAS	P08631	13,14
	LYN (1)	JTK8; regulates growth factor/cytokine/integrin-mediated innate immune responses	P07948	15
	PTK6 (1)	BRK; controls the differentiation and maintenance of normal epithelia & tumor growth	Q13882	16
	SRMS (1)	PTK70; phosphorylates DOK1, KHDRBS1/SAM68 and VIM	Q9H3Y6	-
	ABL1 (1)	Proto-oncogene tyrosine-protein kinase; regulates adhesion, motility & differentiation	P00519	-
	ABL2 (1)	Proto-oncogene tyrosine-Protein kinase; regulates adhesion, motility & differentiation	P42684	-
	BTK (1)	ATK; BPK; XLA; B-cell development & differentiation & signaling	Q06187	17-22
	ITK (1)	LYK; EMT; binds GATA3; regulates T-cell development, function & differentiation	Q08881	23,24
	TEC (1)	PSCTK4; regulates the development, function and differentiation of diverse cell types	P42680	-
	CRK (2)	CRKII; regulates cell adhesion, spreading & migration	P46108	1,3,25,26
	CRKL (2)	CRK-like adaptor protein that activate the RAS & JUN kinase signaling pathways	P46109	-
	GRAP1 (2)	DFN114; a BCR-ABL binding enzyme involved in RAS signaling pathway	Q13588	27
	GRAP2 (2)	GRID; MONA; binds SHC, GAB1, LCP2, SLP76; involved in NF-AT activation	O75791	-
	GRAPL (1)	Involved in receptor tyrosine kinase binding	Q8TC17	-
	GRB2 (2)	ASH; NCKAP2; binds SHC, GAB1, FRS2, CBL; links surface receptors to RAS signaling	P62993	28-31
	SLA (1)	SLAP1; SLA1; links ZAP70 with CBL & negatively regulates T-cell receptor signaling	Q13239	32,33
	SLA2 (1)	SLAP2; MARS; links ZAP70 with CBL & negatively regulates T-cell receptor signaling	Q9H6Q3	-
	RASA1 (1)	p120RASGAP; CMAVM1; acts as a GAP of RAS	P20936	34
	PLCG1 (1)	PLC1; PLC148; NCKAP3; catalyzes DAG & IP3 production	P19174	35-38
	PLCG2 (1)	PLCIV; APLAID; FCAS3; catalyzes DAG & IP3 production	P16885	-
	MAP3K21 (1)	MLK4; negative regulator of TLR4 signaling	Q5TCX8	-
	TNK2 (1)	ACK1; phosphorylates AKT1, AR, WASP; mediates CDC42-dependent cell migration	Q07912	39,40
	MAP3K10 (1)	MLK2; MST; MEKK10; activates JNK & SEK1 pathways	Q02779	-
	MAP3K11 (1)	MLK3; PTK1; MEKK11; SPRK; activates BRAF, ERK, p38 and JNK1 pathways	Q16584	-
	TNK1 (1)	Negative regulates the RAS-MAPK pathway; utilized broadly during fetal development	Q13470	-
2: BAR and/or PH plus RHOGAP or RHOGEF domains	ARHGAP10 (1)	GRAF2; PSGAP; acts as a GAP on CDC42 & RHOA; involved in actin organization	A1A4S6	41
	ARHGAP26 (1)	GRAF1; acts as a GAP on RHO family proteins in pathways related focal adhesion	Q9UNA1	41-43
	ARHGAP42 (1)	GRAF3; acts as a GAP on RHO family proteins in vascular smooth muscle	A6NI28	-
	ASAP1 (1)	AMAP1; Centaurinβ4; ARF1/ARF5GAP; coordinate membrane trafficking; ciliogenesis	Q9ULH1	44-47
	ASAP2 (1)	AMAP2; Centaurinβ3; ARFGAP; PYK2 & SRC substrate; regulates vesicular transport	O43150	-
	ARHGAP27 (1)	CAMGAP1; SH3D20; acts as a GAP on RHO family proteins in endocytosis	Q6ZUM4	-
	ARHGAP9 (1)	RGL1; acts as a CDC42/RAC1 GAP; regulates matrix adhesion of hematopoietic cells	Q9BRR9	-
	ARHGAP12 (1)	Acts as a GAP on RHO family proteins, maybe downstream of the GPCR Signaling	Q8IWW6	48
	SKAP1 (1)	SCAP1; SKAP55; positively regulates T-cell receptor and promotes the MAPK pathway	Q86WV1	49
	SKAP2 (1)	SCAP2; PRAP; SAPS; involved in B-cell and macrophage adhesion processes	O75563	50-53
	ARHGEF26 (1)	SGEF; RHOGGEF; macropinocytosis; trans-endothelial migration of leukocytes	Q96DR7	-
	NGEF (1)	ARHGEF27; EPHEXIN1; involved in ephrin-induced axon & spine morphogenesis	Q8N5V2	-
	ARHGEF19 (1)	Ephexin2; WGEF; RHOAGEF; interacts with BRAF & activates MAPK pathway	Q8IW93	-
	ARHGEF16 (1)	Ephexin4; RHOG/CDC42GEF; cell migration	Q5VV41	-
	ARHGEF5 (1)	Ephexin-3; p60TIM; RHOAGEF; involved in SRC-induced podosome formation	Q12774	-
	ARHGEF4 (1)	ASEF1; STM6; a CDC42 GEF, involved in cell-cell adhesion & migration	Q9NR80	54-56
	SPATA13 (1)	ASEF2; ARHGEF29; acts as a CDC42GEF in cell migration & adhesion	Q96N96	54-56
	ARHGEF9 (1)	HPEM1; Collybistin; RAC/CDC42GEF; formation of GABAergic & glycinergic synapses	O43307	-
	OBSCN (1)	ARHGEF30; Obscurin; a giant sarcomeric protein; calmodulin and titin binding	Q5VST9	57
	ARHGEF6 (1)	αPIX; COOL2; associated with X-linked intellectual disability	Q15052	58-60
	ARHGEF7 (1)	βPIX, CCOL1; cell adhesion, spreading & migration	Q14155	61-63
	TRIO (2)	ARHGEF23; MRD44; acts as a dual RAC1/RHOAGEF in hippocampal neurons	O75962	-
	KALRN (2)	Kalirin; ARHGEF24; DUO; TRAD; regulates as a RHOGEF neuronal growth & plasticity	O60229	-
	MCF2L (1)	ARHGEF14; RHOA/CDC42GEF associated with osteoarthritis	O15068	-
	VAV1 (2)	Acts as a RAC1/RHOAGEF; involved in cell differentiation & proliferation	P15498	30,31,64,65
	VAV2 (2)	Acts as a RAC1GEF; involved in angiogenesis & endothelial cell migration	P52735	-
	VAV3 (2)	Acts a RHOA/RHOGGEF; involved in angiogenesis & endothelial cell migration	Q9UKW4	-

3: Several or single SH3 plus other shared domains	ABI1 (1)	E3B1; binds Abl, spectrin & EPS8; regulate the dendritic outgrowth & branching	Q8IZP0	3,26 66,67
	ABI2 (1)	ArgBP1; component of the WAVE complex; involved in cell motility & adhesion	Q9NYB9	68
	ABI3 (1)	NESH; component of the WAVE complex, regulates dendritic Spine Morphology	Q9P2A4	69
	BAIAP2L1 (1)	IRTKS; IR substrate; RAC1 binding; promotes actin assembly & membrane protrusions	Q9UHR4	70-72
	BAIAP2L2 (1)	Pinkbar; formation of curved membrane structures	Q6UXY1	-
	BAIAP2 (1)	IRS58; links RAC1/CDC42 to downstream effectors; promotes filopodial protrusions	Q9UQB8	-
	RUSC1 (1)	NESCA; regulates MAPK & NFκB pathways, & NGF-dependent neurite outgrowth	Q9BVN2	-
	RUSC2 (1)	MRT61; IPORIN; acts as a RAB35 effector on intracellular vesicular trafficking	Q8N2Y8	-
	SH3YL1 (1)	RAY; involved in hair follicle development, cell migration, & dorsal ruffle formation	Q96HL8	73,74
	PEX13 (1)	PEROXIN13; NALD; involved in the import of peroxisomal biogenesis factors PTS1/2	Q92968	75,76
	MAPK8IP1 (1)	JIP1; IB1; involved as MAPK component in survival response	Q9UQF2	77
	MAPK8IP2 (1)	JIP2; IB2; involved as MAPK component in survival response	Q13387	-
	FUT8 (1)	CDGF1; a Golgi associated enzyme regulates adhesion, migration & invasion	Q9BYC5	-
	EFS (1)	HEFS, CAS3; SIN; acts as SRC activator on cell adhesion	O43281	78,79
	DBNL (1)	SH3P7; ABP1; CMAP; HIP55; involved endocytic pathways & podosome formation	Q9UJU6	-
	PPP1R13B (1)	ASPP1; regulates the DNA binding & transactivation function of p53	Q96KQ4	80,81
	TP53BP2 (1)	ASPP2; P53BP2; regulates cell growth & apoptosis by binding to p53 & BCL2	Q13625	80-82
	PPP1R13L (1)	IASPP; NKIP1; RIA4; inhibits p53 & NFκB; regulates apoptosis and transcription	Q8WUF5	-
	OSTF1 (1)	SH3P2; OSF; induces bone resorption & enhances osteoclast formation & activity	Q92882	83
	BIN1 (1)	AMPHL; SH3P9; membrane curvature & remodeling; negative regulator of endocytosis	O00499	84-87
	GAS7 (1)	KIAA0394; promotes maturation & morphological differentiation of cerebellar neurons	O60861	-
	AMPH (1)	Amphiphysin; involved in regulated endocytosis	P49418	88
	Endophilin A2 (1)	SH3GL1; SH3D2B; acts on membrane shaping & clathrin-independent endocytosis	Q99961	-
	Endophilin B2 (1)	SH3GLB2; RRIG1; involved in endocytosis	Q9NR46	-
	Endophilin B1 (1)	SH3GLB1; BIF1; involved in membrane fusion & in the regulation of autophagy	Q9Y371	-
	Endophilin 1 (1)	SH3GL2; SH3D2A; acts on membrane shaping & synaptic vesicle endocytosis	Q99962	-
	Endophilin 3 (1)	SH3GL3; SH3D2C; implicated in membrane shaping & endocytosis	Q99963	89
	STAC (2)	Involved in the modulation of calcium channel at the cell membrane	Q99469	-
	STAC2 (2)	24B2; involved in the modulation of calcium channel at the cell membrane	Q6ZMT1	-
	STAC3 (2)	MYPBB; NAM; Required for excitation-contraction coupling in skeletal muscle	Q96MF2	90
	DNMBP (6)	TUBA; ARHGEF36; links dynamin to actin regulatory proteins & is involved in adhesion	Q6XZF7	91-94
	ARHGEF37 (2)	FLJ41603; RHOGEF; clathrin-mediated endocytosis, GPCR & p75-NRT signaling	A1IGU5	-
	ARHGEF38 (2)	FLJ20184; RHOGEF; GPCR & p75-NRT signaling	Q9NXL2	-
	SH3BP4 (2)	EHB10; TTP; BOG25; controls clathrin-mediated endocytosis	Q9P0V3	95,96
	TSPOAP1 (3)	RIMBP1; RBP1; PRAX1; synchronizes and couples synaptic vesicle to the exocytic sites	O95153	97,98
	SH3PXD2A (5)	TKS5; SH3MD1; involved in ROS generation, podosome formation & ECM degradation	Q5TCZ1	99
	SH3PXD2B (4)	TSK4; FAD49; involved in ROS generation, podosome formation & ECM degradation	A1X283	99
	SH3RF1 (4)	POSH1; SH3MD2; involved in dynamin-dependent endocytosis & JNK activation	Q7Z6J0	100
	SH3RF3 (4)	POSH2; SH3MD4; is a RAC effector & mediates proteasomal degradation	Q8TEJ3	101,102
	SH3D19 (5)	EBP; EVE-1; acts on ADAMs/EGFR axis & suppresses RAS-induced cell transformation	Q5HYK7	-
	ITSN1 (5)	SH3D1A; SH3P17; acts as a CDC42GEF on actin nucleation & endocytosis	Q15811	103-109
	ITSN2 (5)	SH3P18; SWAP; acts as a CDC42GEF on actin nucleation & endocytosis	Q9NZM3	-
	CD2AP (3)	CMS; involved in receptor clustering & cytoskeletal polarity	Q9Y5K6	47,110
	NCK1 (3)	Acts as an RTK-associated protein on RAS signaling & dsRNA-induced PKR activation	P16333	63,111,112
	NCK2 (3)	GRB4; acts as an RTK-associated protein on RAS signaling & translational initiation	O43639	111
	RIMBP3B (3)	Plays a key role in sperm head morphogenesis during late stages of sperm development	A6NNM3	-
	RIMBP3C (3)	Plays a key role in sperm head morphogenesis during late stages of sperm development	A6NJZ7	-
	RIMBP3 (3)	RIMBP3A; plays a key role in sperm head morphogenesis during sperm development	Q9UFD9	-
	TSPOAP1 (3)	RIMBP1; RBP1; PRAX1; synchronizes and couples synaptic vesicle to the exocytic sites	O95153	97,98
	RIMBP2 (3)	RBP2; PPP1R133; synchronizes and couples synaptic vesicle to the sites of exocytosis	O15034	-
	SH3KBP1 (3)	CD2BP3; CIN85; HSB1; controls cell shape & migration, & stimulates B cell activation	Q96B97	-
	SH3RF2 (3)	POSH3; HEPP1; mediates TNFα signaling & proteasomal degradation	Q8TEC5	113
	SORBS1 (3)	SH3P12; FLAF2; CAP; involved in formation of actin stress fibers and focal adhesions	Q9BX66	114
	SORBS2 (3)	ARGBP2; forms complex with ABL1/CBL & promotes ABL1 ubiquitination & degradation	O94875	-
	SORBS3 (3)	VINEXIN; SH3D4; SCAM1; plays a role in cell spreading	O60504	115,116
4: PDZ and/or GuaKin domain	MPP2 (1)	DLG2; negatively regulates SRC function in epithelial cells	Q14168	117
	PALS2 (1)	MPP6; VAM1; act on receptor clustering by forming multiprotein complexes	Q9NZW5	-
	PALS1 (1)	MPP5; involved in adherens junction biogenesis & localization of the exocyst complex	Q8N3R9	-
	MPP3 (1)	DLG3; interact with the cytoskeleton & regulates intracellular junctions & cell proliferation	Q13368	-
	MPP7 (1)	Promotes epithelial cell polarity and tight junction formation	Q5T2T1	-
	CASK (1)	CSKP; FGS4; LIN2; HCASK; a Ca2+/CAM-dependent kinase involved in neurogenesis	O14936	-
	MPP4 (1)	DLG6; plays a role in retinal photoreceptors development.	Q96JB8	-
	MPP1 (1)	EMP55; AAG12; EMP55; as a MAGUK family proteins regulates neutrophil polarity	Q00013	-
	DLG3 (1)	MRX90; SAP102; XLMR; involved in NMDA receptor-mediated synaptic plasticity	Q92796	-
	DLG4 (1)	PSD95; SAP90; required for synaptic plasticity associated with NMDA receptor signaling	P78352	-
	DLG1 (1)	SAP97; DLGH1; involved in synaptogenesis & lymphocyte activation	Q12959	118
	DLG2 (1)	PSD93; binds NMDA receptor subunits & regulates excitatory synapses	Q15700	119
	DLG5 (1)	PDLG; involved in dendritic spine formation & synaptogenesis as well as ciliogenesis	Q8TDM6	-
	TJP1 (1)	ZO1; involved in tight junction organization, epithelial polarization and barrier formation	Q07157	120
	TJP2 (1)	ZO2; PFIC4; DFNA51; plays a role in tight junctions and adherents junctions	Q9UDY2	-
	TJP3 (1)	ZO3; links tight junction transmembrane proteins	O95049	-
	CACNB2 (1)	CACNLB2; CAVB2; MYSB; a subunit of voltage-dependent calcium channels	Q08289	121
	CACNB1 (1)	CACNLB1; CAB1; CCHLB1; regulates the activity of L-type calcium channels	Q02641	-

	CACNB3 (1)	CACNLB3; CAB3; a regulatory subunit of the voltage-gated calcium channel	P54284	-
	CACNB4 (1)	CACNLB4; CAB4, EJM4; a dihydropyridine-sensitive subunit of L-type calcium channel	O00305	-
	SHANK2 (1)	CORTBP1; involved in structural and functional organization of the dendritic spine	Q9UPX8	-
	SHANK1 (1)	SSTRIP; acts in GKAP/PSD95/HOMER complex on dendritic spine organization	Q9Y566	122
	SHANK3 (1)	PSAP2; acts on th dendritic spine and synapse formation, maturation and maintenance	Q9BYB0	123
5: FCH and/or RHOGAP domains	FCHSD1 (2)	NWK2; promotes SNX9WASL-mediated actin polymerization.	Q86WN1	-
	FCHSD2 (2)	NWK1; SH3MD3; promotes actin polymerization & internalization of surface receptors	O94868	-
	FNBP1 (1)	FBP17; Rapostlin; links RND2 signaling to F-actin & spine morphogenesis	Q96RU3	-
	NOSTRIN (1)	Multivalent adapter protein involved in NO metabolism by sequestering NOS3	Q8IVI9	124-126
	TRIP10 (1)	CIP4; STP; promotes CDC42(WASP-induced actin polymerization	Q15642	127,128
	FNBP1L (1)	TOCA1; binds CDC42/WASP; promote membrane tubulation & F-actin reorganization	Q5T0N5	-
	PACSIN1 (1)	SYNDAPIN1; recruits DNM1/2/3 to membranes; regulates neurite formation & branching	Q9BY11	129
	PACSIN2 (1)	SYNDAPIN2; involved in plasma membrane protein internalization by endocytosis	Q9UNF0	129
	PACSIN3 (1)	SYNDAPIN3; involved in cell-surface receptor internalization by endocytosis	Q9UKS6	129
	PSTPIP1 (1)	CD2BP1L; PAPAS; regulates WAS actin-bundling activity, endocytosis and cell migration	O43586	130-133
	PIK3R1 (1)	p85α; AMG7; regulates membrane binding & activity of p110 catalytic subunit of PI3K	P27986	134-137
	PIK3R2 (1)	P85β; MPPH1; regulates membrane binding & activity of p110 catalytic subunit of PI3K	O00459	136
	ARHGAP32 (1)	p200; GRIT; acts as a RHOGAP in the differentiation of neuronal cells	A7KAX9	-
	ARHGAP33 (1)	SNX26; TCGAP; acts as a GAP on RHO family proteins in intracellular trafficking	O14559	-
	ARHGAP4 (1)	RGC1; SRGAP4; acts as RHOGAP in hematopoietic cells	P98171	-
	SRGAP3 (1)	ARHGAP14; WRP; MEGAP; WAVE-associated Rac1/CDC42GAP	O43295	138,139
	SRGAP1 (1)	ARHGAP13; Acts as RHOA/CDC42GAP in neuronal migration	Q7Z6B7	138
	SRGAP2 (1)	ARHGAP34; regulates as a RAC1GAP cell migration and differentiation	O75044	140
6: UBA & HPhos	UBASH3A (1)	TULA1, STS2; as a T-cell ubiquitin ligand family member negatively act on T-cell signaling	P57075	141-144
	UBASH3B (1)	TULA2; STS1; as a T-cell ubiquitin ligand family member negatively act on T-cell signaling	Q8TF42	143,144
7: S-rich & CAS-C	CASS4 (1)	CAS4; HEFL; regulates focal adhesion integrity & cell spreading	Q9NQ75	-
	BCAR1 (1)	p130CAS; CAS1; CASS1; regulates cell adhesion & migration	P56945	1-3,25,145-147
	NEDD9 (1)	CAS2; CASL; CASS2; regulates cell adhesion & migration	Q14511	148,149
8: Myosin &/or MyTH4	MYO7A (1)	DFNB2; NSRD2; mediates in complex with USH1C/G & CDH23 mechanotransduction	Q13402	-
	MYO7B (1)	MYOVIIb; acts in the intermicrovillar adhesion complex on microvilli organization & length	Q6PIF6	-
	MYO15A (1)	DFNB3; unconventional MYO15 required for stereocilia formation in mature hair bundles.	Q9UKN7	-
	MYO15B (1)	MYO15BP; no functional motor domain	Q96JP2	-
	MYO1E (1)	FSGS6; HUNCM-IC; controls the movement of class II-containing cytoplasmic vesicles	Q12965	150-152
	MYO1F (1)	Acts with MYO1E on innate immunity in cell migration & phagocytosis	O00160	-
9: SAM* along with PTB and SLY	EPS8L1 (1)	DRC3; EPS8R1; involved in membrane ruffling & remodeling of the actin cytoskeleton	Q8TE68	112
	EPS8L2 (1)	DFNB106; required for stereocilia maintenance in adult hair cells	Q9H6S3	-
	EPS8L3 (2)	EPS8R3; function unknown	Q8TE67	-
	EPS8 (1)	DFNB102; regulates in complex with SOS1/ABI1 cell migration & invasion	Q12929	67,153-155
	SASH1 (1)	PEPE1; SH3D6A; Acts on TLR4/NFκB signaling & LPS-induced endothel. cell migration	O94885	156
	SAMSN1 (1)	HACS1; SH3D6B; acts on RAC1-dependent cell spreading & polarization	Q9NSI8	157
	SASH3 (1)	HACS2; SH3D6C; functions as a signaling adapter protein in lymphocytes	O75995	-
	CASKIN1 (1)	CSKI1; ANKS5A; links CASK to downstream intracellular effectors	Q8WXD9	-
	CASKIN2 (1)	CSKI2; ANKS5B; links CASK to downstream intracellular effectors	Q8WXE0	-
10: Diverse domains	NCF1B (2)	NCF1B; required for activation of the latent NADPH oxidase	A6NI72	-
	NCF1C (2)	NCF1C; required for activation of the latent NADPH oxidase	A8MVU1	-
	NCF1 (2)	p47phox; NOXO2; NCF1A; required for activation of the latent NADPH oxidase	P14598	158-160
	NCF4 (1)	p40phox; SH3PXD4; involved assembly & activation of the NADPH oxidase complex	Q15080	158-160
	NOXO1 (2)	SH3PXD5; p41NOX; activates together with NOX2 NOX1/3	Q8NFA2	158,161
	SNX18 (1)	SNAG1; SH3PX2; stimulates DNM2 GTPase activity; involved in endocytosis	Q96RF0	162,163
	SNX9 (1)	SH3PX1; SDP1; WISP; stimulates DNM2 GTPase activity; involved in endocytosis	Q9Y5X1	164-173
	SNX33 (1)	SH3PX2; reorganizes the cytoskeleton, endocytosis and cellular vesicle trafficking	Q8WV41	-
	NCF2 (2)	p67phox; NOXA2; required for activation of the latent NADPH oxidase	P19878	158-160
	SH3TC2 (2)	CMT4C; MNMN; involved as a RAB11 effector in axoglial interactions myelination	Q8TF17	174,175
	NOXA1 (1)	p51NOX; activates as a p67^{phox}-like factor NOX1/3 in the host defense & oxygen sensing	Q86UR1	158,161
	SH3TC1 (1)	Unknown function	Q8TE82	-
	NEBL (1)	LASP2; LIM-Nebulette; links sarcomeric actin to desmin around the Z-disk	O76041	176
	NEB (1)	NEBU; NEM2; NEB177B; binds & stabilize F-actin; involved in sarcomeric integrity	P20929	177
	LASP1 (1)	MLN50; regulates actin-associated ion transport activities	Q14847	176,178-182
	AHI1 (1)	JBTS3; involved in vesicle trafficking, ciliogenesis & WNT signaling	Q8N157	183
	PRMT2 (1)	ANM2; methylates arginines in STAT3, FBL, & H4; involved in growth regulation	P55345	184
	FYB (1)	SLAP130; THC3; binds FYN and LCP2 & regulates actin cytoskeleton in T-cells	O15117	49
	HCLS1 (1)	LCKBP1; HS1; CTTNL; involves in antigen receptor signaling in lymphoid cells	P14317	-
	CTTN (1)	SRC8; EMS1; Amplaxin; involved in the formation of lamellipodia and in cell migration	Q14247	-
	MACC1 (1)	SH3BP4L; 7A5; promotes HGF-MET signaling & cell motility, proliferation & metastasis	Q6ZN28	185,186
	MAP3K9 (1)	MLK1; MEKK9; activates JNK pathway; involved in the cytochrome-C release & apoptosis	P80192	-
	SGSM3 (1)	MAP; RUSC3; RABGAP5; involved in NF2-mediated growth suppression of cells	Q96HU1	-
	NCKIPSD (1)	SPIN90; WISH; WASLBP; stimulates N-WASP-induced ARP2/3 complex activation	Q9NZQ3	4,63,187
	STAM (1)	STAM1; HSE1H; involved in signal transduction mediated by cytokines and growth factors	Q92783	-
	STAM2 (1)	HBP; involved in signal transduction mediated by cytokines and growth factors	O75886	-

11: Spectrin and EF-hand domain	SPTA1 (1)	SPH3; EL2; forms the cytoskeletal superstructure of the erythrocyte plasma membrane	P02549	-
	SPTAN1 (1)	NEAS; EIEE5; involved in calcium-dependent cytoskeleton movement at the membrane	Q13813	188
	DSP (1)	DESP; Desmoplakin; is part of the desmosomal cadherin-plakoglobin complexes	P15924	-
	DST (1)	Dystonin; BPAG1; MACF2; acts as a cytoskeletal linker protein on axonal transport	Q03001	-
	MACF1 (1)	ACF7, LIS9; OFC4; involved in AXIN1/APC/CTNNB1/GSK3B complex translocation	Q9UPN3	-
12: DOCK & DHR domains	DOCK2 (1)	IMD40; involved as RAC1/2 GEF in lymphocyte migration	Q92608	189,190
	DOCK4 (1)	KIAA0716; with its RHOGEF function regulates cell migration	Q8N1I0	191
	DOCK1 (1)	DOCK180; as a GEF regulates cell spreading & migration	Q14185	3,25
	DOCK3 (1)	MOCA; PBP; activates as a RACGEF the WAVE complex & induces axonal outgrowth	Q8IZD9	-
	DOCK5 (1)	Associates with CRK/CRKL, & regulates epithelial cell spreading & migration	Q9H7D0	-
13: single SH3 domain	FYB2 (1)	ARAP; T-cell receptor signaling & integrin-mediated adhesion	Q5VWT5	-
	MIA (1)	MIA1; Associated with melanoma, glioma and neuroectodermal tumors	Q16674	
	MIA2 (1)	MGEA11; TALI; MEA6; involved in cholesterol & TAG homeostasis, & OL7A1 secretion	Q96PC5	-
	MIA3 (1)	TANGO; ARNT; required for membrane-bound ER-resident complexes consisting of MIA2	Q5JRA6	192
	NPHP1 (1)	NPH1; Nephrocystin-1; control together with PTK2B/PYK2 the epithelial cell polarity	O15259	193-195
	OTOR (1)	Otoraplin; MIAL1; FDP; functions in cartilage development and maintenance	Q9NRC9	-
	PRAM (1)	PRAM1; PMLRAR; involved in myeloid differentiation & integrin signaling in neutrophils	Q96QH2	-
	SH3D21 (1)	Unknown function	A4FU49	-

[a]To provide comprehensive alias information and interaction details, Gene Cards database was utilized.
[b]UniProt ID was included to better identify SH3DCPs due to their various names.

Table S2. List of protein domains found in SH3DCP (alphabetical order)

Domains	Full name
2'-5'_RNA_ligase2	2'-5' RNA ligase 2 domain
Abi_alpha	ABL interactor alpha domain
Abi_HHR	ABL interactor homeodomain homologous region
Ank	Ankyrin repeat
ARFGAP	ARF GTPase activating protein
ARHGEF5	RHO guanine nucleotide exchange factor 5
Baculo_p24	Baculovirus P24-like
BAR	Bin/amphiphysin/Rvs
BAR_3_WASP_bdg	BAR 3 domain of WASP interacting protein
betaPIX_CC	BetaPIX coiled-coil domain
BTK	Bruton tyrosine kinase
C1	Protein kinase C conserved region 1
C2	Protein kinase C conserved region 2
CAS_C	CRK-associated substrate C-terminal
Caskin1-CID	CASKIN1 carboxy-terminal interaction domain
Caskin-Pro-rich	CASKIN proline-rich domain
Caskin-tail	CASKIN carboxy-terminal domain
CH	Calponin homology domain
Cofilin_ADF	Cofilin-actin-depolymerizing factor
Cohesin_load	Cohesin loader N-terminal domain
CRAL_TRIO	CRAL-TRIO lipid binding domain
CSD	Cold-shock domain
CSD3_N	Cold shock domain 3, N-terminal domain
CTNNBL	Catenin-beta-like domain
CTV_P33	Citrus tristeza virus P33
CYYR1	Cysteine/tyrosine-rich 1 domain
dbPDZ_assoc	Unstructured region between two PDZ domains on Dlg5 or PDZ domain, Dlg/ZO-1-associated domain
DEDD_Tnp_IS110	Transposase for efficient DNA transposition or DEDD-like exonuclease TnpI-IS110 domain
DegS	Signal transduction histidine kinase DegS
DHR-2_Lobe_A	DHR-2 lobe A domain
DHR-2_Lobe_B	DHR-2 lobe B domain
DHR-2_Lobe_C	DHR-2 lobe C domain
DOCK_N	Dedicator of cytokinesis N-terminal domain
DOCK-C2	DOCK C2 domain
DUF1539	Domain of unknown function 1539
DUF1664	Domain of unknown function 1664
DUF1778	Domain of unknown function 1778
DUF2605	Domain of unknown function 2605
DUF4100	Domain of unknown function 4100
DUF4404	Domain of unknown function 4404
DUF6781	Domain of unknown function 6781
EF-hand	EF-hand calcium-binding domain
EFhand_Ca_insen	Ca^{2+} insensitive EF hand
EF-hand_like	EF-hand-like domain
ERM_helical	Ezrin/radixin/moesin, alpha-helical domain
Exonuc_VII_L	Exonuclease VII large subunit domain
F_actin_bind	F-actin binding domain
FCH	Fes-CIP4 homology
FERM_f0	FERM domain F0 subdomain
FERM_M	FERM domain M subdomain
Filamin	Actin-binding filamin domain
FlaC_arch	Archaeal flagellar accessory protein (FLaCa) domain
Flg_hook	Flagellin hook region
fn3	Fibronectin type III domain
FTZ	Fushi tarazu
FUT8_N_cat	Fucosyltransferase 8 N-terminal and catalytic domains
GAS2	Growth arrest-specific protein 2
GAT	GGA and Tom1 (GAT) domain
GDPD	Glycerophosphodiester phosphodiesterase
Glyco_hyd_101C	Glycosyl hydrolase family 101, subfamily C
GP3_package	Glycoprotein 3 (GP3) packaging domain
GrpE	Nucleotide exchange factor GrpE
GSH_synthase	Glutathione synthetase
GTPase_binding	GTPase binding domain
Guanylate_kin	Guanylate kinase domain
HAUS-augmin3	HAUS augmin-like complex subunit 3
His_Phos	Histidine phosphatase domain
HOIP-UBA	HOIL-1 interacting protein ubiquitin-associated domain
HR1	Homology region 1
HS1_rep	Repeat in HS1/Cortactin
hSH3	Helically-extended SH3 domain

Ig	Immunoglobulin domain
IMD	IRSp53/MIM homology domain
Inhibitor_Mig-6	Mitogen-activated protein kinase (MAPK) inhibitor-6 domain
INTAP	Intersectin and clathrin adaptor AP2 binding region
I-set	Immunoglobulin I-set domain
KxDL	KxDL domain
L27	L27 domain
L27_N	N-Terminal L27 domain
Laminin_II	Laminin Domain II
LANC_like	Lanthionine synthetase C-like protein domain
LIM	Lin-11/Isl-1/Mec-3
LMBR1	LMBR1 domain
Lzipper-MIP1	Leucine-zipper of ternary complex factor MIP1 domains
MAGUK_N_PEST	Membrane-associated guanylate kinase (MAGUK) N-terminal and Polyubiquitination (PEST) domains
MCU	Mitochondrial calcium uniporter domain
Methyltransf	Methyltransferase domain
MIC19_MIC25	Mitochondrial contact site and cristae organizing system (MICOS) subunits MIC19 and MIC25
MTS	Methyltransferase small domain
Myb_DNA-bind	Myb-like DNA-binding domain
Myosin_head	Myosin head domain
Myosin_TH1	Class I myosin tail homology domain
MyTH4	Myosin tail homology 4 (MYTH4) domain
NBCH_WD40	Neurobeachin, beta-propeller domain
Nebulin	Nebulin repeat domain
NECFESHC	SH3 terminal domain of 2nd SH3 on Neutrophil cytosol factor 1
NHS	Nance-Horan syndrome protein (NHS)
NPF	Asn-Pro-Phe domain
OmpH	Outer membrane protein (OmpH-like)
p47_phox_C	NADPH oxidase subunit p47Phox, C terminal domain
PB1	Phox and Bem1 domain
PDZ	PSD-95/Discs large/ZO-1
Peptidase_M1	Peptidase M1 domain
Peptidase_M50	Peptidase M50 domain
Peroxin-13_N	Peroxin-13 N-Terminal domain
PH	Pleckstrin homology domain
PI3K_P85_iSH2	Phosphatidylinositol 3-kinase regulatory subunit P85 inter-SH2 domain
PID	Phosphotyrosine interaction domain
Pilin_GH	Type IV pilin-like G and H, putative
PI-PLC-X	Phosphatidylinositol-specific phospholipase C X-domain
PI-PLC-Y	Phosphatidylinositol-specific phospholipase C Y-domain
PK_Tyr_Ser-Thr	Protein kinase Tyr/Ser/Thr domain
Pkinase	Protein kinase domain
Plectin	Plectin repeat
PTB	Phosphotyrosine binding domain
PX	Phox homology
RABGAP-TBC	RAB GTPase-activating protein TBC domain
RASGAP	RAS GTPase-activating protein
RHOGAP	RHO GTPase-activating protein
RHOGEF	RHO guanine nucleotide exchange factor
RHOGEF67_u1	Unstructured region one on RhoGEF 6 and 7
RHOGEF67_u2	Unstructured region two on RhoGEF 6 and 7
RNase_Y_N	Ribonuclease Y N-terminal domain
RPEL	RPEL (RPxxxEL) motif
RsgA_GTPase	RsgA GTPase
RUN	RPIP8/UNC-14/NESCA
SAM*	Sterile Alpha Motif (denoted as SAM* when regarded as a domain rather than a protein)
SAM_PNT	Sterile alpha motif (SAM)/Pointed domain
Serine_rich	Serine-rich domain
SH2	SRCc homology 2 domain
SH3BP5	SH3 domain-binding protein 5
SLY	SH3-domain containing protein expressed in lymphocytes
SNAP	synaptosome-associated protein 25kDa
Sorb	Sorbin homologous domain
Spectrin	Spectrin repeat
Spectrin_like	Spectrin-like domain
SPIN90_LRD	leucine-rich domain (LRD) within the C-terminal domain of SPIN90 (also known as NCK interacting protein with SH3 domain (NCKIPSD))
STAC2_u1	Unstructured on SH3 and cysteine-rich domain-containing protein 2
Takusan	Takusan (“many” in Japanese) protein family regulates synaptic activity
TcpQ	Toxin co-regulated pilus biosynthesis protein Q
Tetrabrachion	Tetrabrachion, parallel right-handed coiled coil domain
Tmemb_cc2	Transmembrane protein with coiled-coil domains 2
TPR	Tetratricopeptide repeat domain

TPR_MalT	Tetratricopeptide repeat domain-containing protein MALT or MalT-like TPR region
Trypan_PARP	Procyclic acidic repetitive protein (PARP)
UBA	Ubiquitin-associated domain
VGCC_beta4Aa_N	Voltage gated calcium channel subunit beta domain 4Aa N terminal
VHS	Vps27, Hrs, and STAM domain
WD40	WD40 repeat domain
WW	Two tryptophan (W) residues
YL1	YL1 protein domain or Vps72/YL1, C-terminal
Ysc84	Ysc84 actin-binding domain
zf-C3HC4	Zinc finger C3HC4 type (RING finger) domain
zf-C4H2	Zinc finger C4H2 type domain
zf-RING_UBOX	Zinc finger, RING-type
ZU5	Zona occludens protein 5 domain

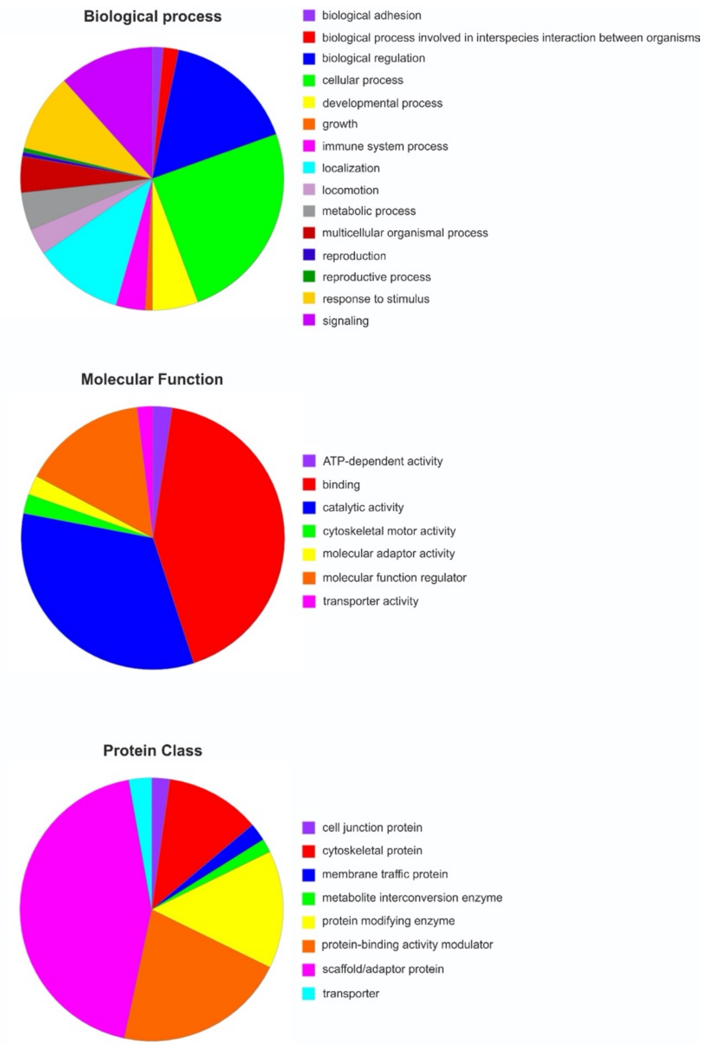

Figure S1. Gene Ontology analysis of the superfamily of human SH3 domain-containing protein. Gene Ontology (GO) terms for the biological process, molecular function, and protein classes of human SH3 domain-containing protein were identified using the PANTHER 17.0 database. In this study, a file including UniProt ID of human SH3 domain-containing proteins were inputted into the PANTHER database. The analysis parameters were set to include Homo sapiens as the species of interest and to retrieve GO terms for functional classification viewed in pie chart. This analysis facilitated a comprehensive understanding of functional characteristics of the human SH3 domain-containing protein superfamily.

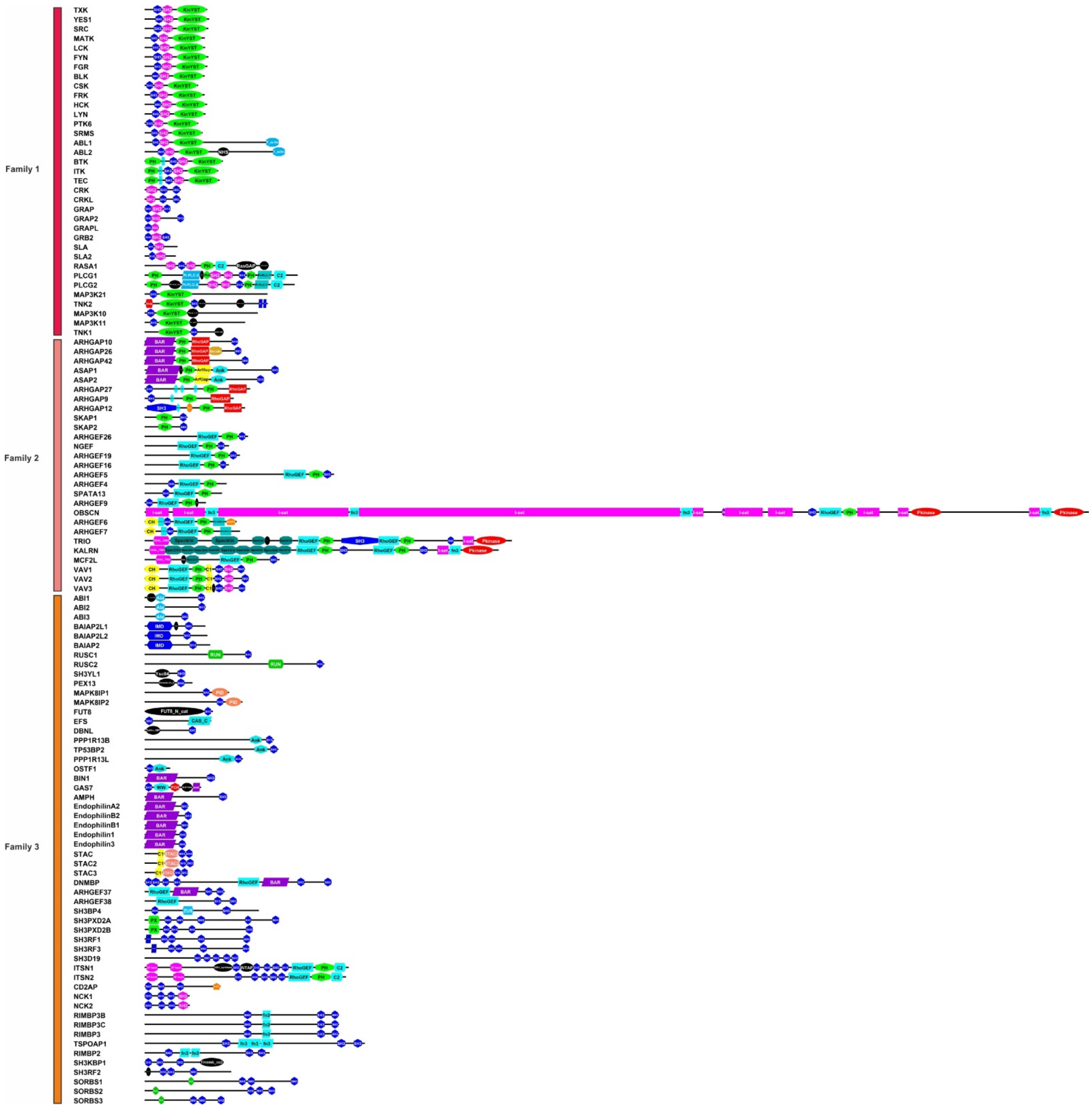
Family 1
Family 2
Family 3
TXK
YES1
SRC
MATK
LCK
FYN
FGR
BLK
CSK
FRK
HCK
LYN
PTK6
SRMS
ABL1
ABL2
BTK
ITK
TEC
CRK
CRKL
GRAP
GRAP2
GRAPL
GRB2
SLA
SLA2
RASA1
PLCG1
PLCG2
MAP3K21
TNK2
MAP3K10
MAP3K11
TNK1
ARHGAP10
ARHGAP26
ARHGAP42
ASAP1
ASAP2
ARHGAP27
ARHGAP9
ARHGAP12
SKAP1
SKAP2
ARHGEF26
NGEF
ARHGEF19
ARHGEF16
ARHGEF5
ARHGEF4
SPATA13
ARHGEF9
OBSCN
ARHGEF6
ARHGEF7
TRIO
KALRN
MCF2L
VAV1
VAV2
VAV3
ABI1
ABI2
ABI3
BAIAP2L1
BAIAP2L2
BAIAP2
RUSC1
RUSC2
SH3YL1
PEX13
MAPK8IP1
MAPK8IP2
FUT8
EFS
DBNL
PPP1R13B
TP53BP2
PPP1R13L
OSTF1
BIN1
GAS7
AMPH
EndophilinA2
EndophilinB2
EndophilinB1
Endophilin1
Endophilin3
STAC
STAC2
STAC3
DNMBP
ARHGEF37
ARHGEF38
SH3BP4
SH3PXD2A
SH3PXD2B
SH3RF1
SH3RF3
SH3D19
ITSN1
ITSN2
CD2AP
NCK1
NCK2
RIMBP3B
RIMBP3C
RIMBP3
TSPOAP1
RIMBP2
SH3KBP1
SH3RF2
SORBS1
SORBS2
SORBS3

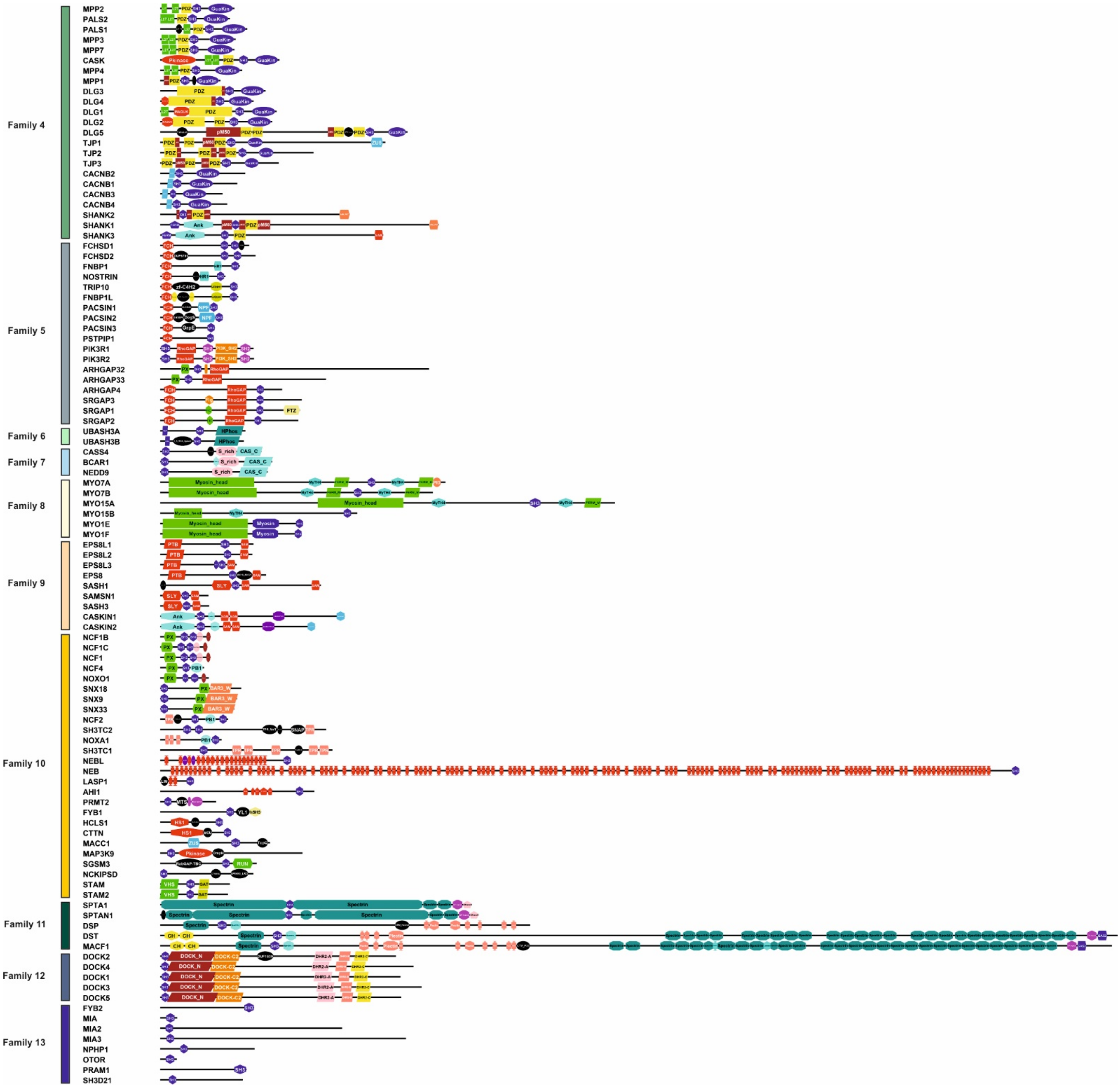

Figure S2. Domain organization of the SH3DCP superfamily. The SH3DCP families are organized based on the phylogenetic tree (Figure 3). Detailed information on all SH3DCPs and their respective domains can be found in Table S1.

References

1. Wallez Y, Mace PD, Pasquale EB, Riedl SJ. NSP-CAS protein complexes: emerging signaling modules in cancer. Genes & cancer. 2012;3(5-6):382-393.
2. Gemperle J, Hexnerová R, Lepšík M, et al. Structural characterization of CAS SH3 domain selectivity and regulation reveals new CAS interaction partners. Scientific reports. 2017;7(1):1-18.
3. Birge RB, Kalodimos C, Inagaki F, Tanaka S. Crk and CrkL adaptor proteins: networks for physiological and pathological signaling. Cell Communication and Signaling. 2009;7(1):1-23.
4. Rönty M, Taivainen A, Heiska L, et al. Palladin interacts with SH3 domains of SPIN90 and Src and is required for Src-induced cytoskeletal remodeling. Experimental Cell Research. 2007/07/15/ 2007;313(12):2575-2585.
5. Ortiz MA, Mikhailova T, Li X, Porter BA, Bah A, Kotula L. Src family kinases, adaptor proteins and the actin cytoskeleton in epithelial-to-mesenchymal transition. Cell Communication and Signaling. 2021/06/30 2021;19(1):67.
6. Silva CM. Role of STATs as downstream signal transducers in Src family kinase-mediated tumorigenesis. Oncogene. 2004/10/01 2004;23(48):8017-8023.
7. Rudd ML, Tua-Smith A, Straus DB. Lck SH3 domain function is required for T-cell receptor signals regulating thymocyte development. Molecular and cellular biology. 2006;26(21):7892-7900.
8. Umemori H, Satot S, Yagi T, Aizawal S, Yamamoto T. Initial events of myelination involve Fyn tyrosine kinase signalling. Nature. 1994;367(6463):572-576.
9. Klein C, Krämer E-M, Cardine A-M, Schraven B, Brandt R, Trotter J. Process outgrowth of oligodendrocytes is promoted by interaction of fyn kinase with the cytoskeletal protein tau. Journal of Neuroscience. 2002;22(3):698-707.
10. Matrone C, Petrillo F, Nasso R, Ferretti G. Fyn tyrosine kinase as harmonizing factor in neuronal functions and dysfunctions. International journal of molecular sciences. 2020;21(12):4444.
11. Lau DH, Hogseth M, Phillips EC, et al. Critical residues involved in tau binding to fyn: implications for tau phosphorylation in Alzheimer’s disease. Acta neuropathologica communications. 2016;4(1):1-13.
12. Annerén C, Lindholm CK, Kriz V, Welsh M. The FRK/RAK-SHB signaling cascade: a versatile signal-transduction pathway that regulates cell survival, differentiation and proliferation. Current molecular medicine. 2003;3(4):313-324.
13. Stanglmaier M, Warmuth M, Kleinlein I, Reis S, Hallek M. The interaction of the Bcr-Abl tyrosine kinase with the Src kinase Hck is mediated by multiple binding domains. Leukemia. 2003;17(2):283-289.
14. Radha V, Sudhakar C, Ray P, Swarup G. Induction of cytochrome c release and apoptosis by Hck-SH3 domain-mediated signalling requires caspase-3. Apoptosis. 2002;7(3):195-207.
15. Hammond S, Wagenknecht-Wiesner A, Veatch SL, Holowka D, Baird B. Roles for SH2 and SH3 domains in Lyn kinase association with activated FcεRI in RBL mast cells revealed by patterned surface analysis. Journal of structural biology. 2009;168(1):161-167.
16. Zheng Y, Peng M, Wang Z, Asara JM, Tyner AL. Protein tyrosine kinase 6 directly phosphorylates AKT and promotes AKT activation in response to epidermal growth factor. Molecular and cellular biology. 2010;30(17):4280-4292.
17. Xue C, Wang X, Zhang L, Qu Q, Zhang Q, Jiang Y. Ibrutinib in B-cell lymphoma: single fighter might be enough? Cancer Cell International. 2020;20(1):1-13.
18. Kueffer LE, Joseph RE, Andreotti AH. Reining in BTK: interdomain interactions and their importance in the regulatory control of BTK. Frontiers in Cell and Developmental Biology. 2021;9:655489.
19. López-Herrera G, Vargas-Hernández A, González-Serrano ME, et al. Bruton's tyrosine kinase—an integral protein of B cell development that also has an essential role in the innate immune system. Journal of leukocyte biology. 2014;95(2):243-250.
20. Cory G, MacCarthy-Morrogh L, Banin S, et al. Evidence that the Wiskott-Aldrich syndrome protein may be involved in lymphoid cell signaling pathways. The Journal of Immunology. 1996;157(9):3791-3795.
21. Nore BF, Mohamed AJ, Vargas L, et al. The Role of Bruton’s Tyrosine Kinase (Btk) in Phosphoinositide-Dependent Signaling. ACI International. 2000;12:3.
22. Akinleye A, Chen Y, Mukhi N, Song Y, Liu D. Ibrutinib and novel BTK inhibitors in clinical development. Journal of hematology & oncology. 2013;6(1):1-9.
23. Andersen TCB, Kristiansen PE, Huszenicza Z, et al. The SH3 domains of the protein kinases ITK and LCK compete for adjacent sites on T cell–specific adapter protein. Journal of Biological Chemistry. 2019;294(42):15480-15494.
24. Perez-Villar JJ, O'Day K, Hewgill DH, Nadler SG, Kanner SB. Nuclear localization of the tyrosine kinase Itk and interaction of its SH3 domain with karyopherin α (Rch1α). International immunology. 2001;13(10):1265-1274.
25. Lawson CD, Ridley AJ. Rho GTPase signaling complexes in cell migration and invasion. Journal of Cell Biology. 2018;217(2):447-457.
26. Hossain S, Dubielecka PM, Sikorski AF, Birge RB, Kotula L. Crk and ABI1: binary molecular switches that regulate abl tyrosine kinase and signaling to the cytoskeleton. Genes & cancer. 2012;3(5-6):402-413.
27. Feng G-S, Ouyang Y-B, Hu D-P, Shi Z-Q, Gentz R, Ni J. Grap Is a Novel SH3-SH2-SH3 Adaptor Protein That Couples Tyrosine Kinases to the Ras Pathway (∗). Journal of Biological Chemistry. 1996;271(21):12129-12132.
28. Schlessinger J. SH2/SH3 signaling proteins. Current opinion in genetics & development. 1994;4(1):25-30.
29. Kazemein Jasemi NS, Herrmann C, Magdalena Estirado E, et al. The intramolecular allostery of GRB2 governing its interaction with SOS1 is modulated by phosphotyrosine ligands. Biochemical Journal. 2021;478(14):2793-2809.
30. Boomer JS, Green JM. An enigmatic tail of CD28 signaling. Cold Spring Harb Perspect Biol. Aug 2010;2(8):a002436.

31. Nishida M, Nagata K, Hachimori Y, et al. Novel recognition mode between Vav and Grb2 SH3 domains. Embo j. Jun 15 2001;20(12):2995-3007.
32. Marton N, Baricza E, Érsek B, Buzás EI, Nagy G. The Emerging and Diverse Roles of Src-Like Adaptor Proteins in Health and Disease. Mediators Inflamm. 2015;2015:952536.
33. Sosinowski T, Pandey A, Dixit VM, Weiss A. Src-like adaptor protein (SLAP) is a negative regulator of T cell receptor signaling. The Journal of experimental medicine. 2000;191(3):463-474.
34. Lapinski PE, King PD. RASA1. In: Choi S, ed. Encyclopedia of Signaling Molecules. New York, NY: Springer New York; 2012:1601-1605.
35. Wang Y, Wu J, Wang Z. Akt binds to and phosphorylates phospholipase C-γ1 in response to epidermal growth factor. Molecular biology of the cell. 2006;17(5):2267-2277.
36. Rellahan BL, Graham LJ, Tysgankov AY, et al. A dynamic constitutive and inducible binding of c-Cbl by PLCγ1 SH3 and SH2 domains (negatively) regulates antigen receptor-induced PLCγ1 activation in lymphocytes. Experimental cell research. 2003;289(1):184-194.
37. Karpov OA, Fearnley GW, Smith GA, et al. Receptor tyrosine kinase structure and function in health and disease. AIMS Biophysics. 2015;2(4):476-502.
38. Chen D, Simons M. Emerging roles of PLCγ1 in endothelial biology. Science signaling. 2021;14(694):eabc6612.
39. Mahajan K, Mahajan NP. ACK1/TNK2 tyrosine kinase: molecular signaling and evolving role in cancers. Oncogene. 2015;34(32):4162-4167.
40. Mahajan K, Mahajan NP. Shepherding AKT and androgen receptor by Ack1 tyrosine kinase. Journal of cellular physiology. 2010;224(2):327-333.
41. Lucken-Ardjomande Häsler S, Vallis Y, Pasche M, McMahon HT. GRAF2, WDR44, and MICAL1 mediate Rab8/10/11–dependent export of E-cadherin, MMP14, and CFTR ΔF508. Journal of Cell Biology. 2020;219(5).
42. Lee S, Salazar SV, Cox TO, Strittmatter SM. Pyk2 signaling through Graf1 and RhoA GTPase is required for amyloid-β oligomer-triggered synapse loss. Journal of Neuroscience. 2019;39(10):1910-1929.
43. Doherty GJ, Åhlund MK, Howes MT, et al. The endocytic protein GRAF1 is directed to cell-matrix adhesion sites and regulates cell spreading. Molecular biology of the cell. 2011;22(22):4380-4389.
44. Bharti S, Inoue H, Bharti K, et al. Src-dependent phosphorylation of ASAP1 regulates podosomes. Molecular and cellular biology. 2007;27(23):8271-8283.
45. Brown MT, Andrade J, Radhakrishna H, Donaldson JG, Cooper JA, Randazzo PA. ASAP1, a phospholipid-dependent arf GTPase-activating protein that associates with and is phosphorylated by Src. Molecular and cellular biology. 1998;18(12):7038-7051.
46. Liu Y, Loijens JC, Martin KH, Karginov AV, Parsons JT. The association of ASAP1, an ADP ribosylation factor-GTPase activating protein, with focal adhesion kinase contributes to the process of focal adhesion assembly. Molecular biology of the cell. 2002;13(6):2147-2156.
47. Liu Y, Yerushalmi GM, Grigera PR, Parsons JT. Mislocalization or reduced expression of Arf GTPase-activating protein ASAP1 inhibits cell spreading and migration by influencing Arf1 GTPase cycling. Journal of Biological Chemistry. 2005;280(10):8884-8892.
48. Ba W, Selten MM, Van Der Raadt J, et al. ARHGAP12 functions as a developmental brake on excitatory synapse function. Cell reports. 2016;14(6):1355-1368.
49. Dadwal N, Mix C, Reinhold A, et al. The multiple roles of the cytosolic adapter proteins ADAP, SKAP1 and SKAP2 for TCR/CD3-mediated signaling events. Frontiers in immunology. 2021;12:703534.
50. Liu J, Kang H, Raab M, da Silva AJ, Kraeft SK, Rudd CE. FYB (FYN binding protein) serves as a binding partner for lymphoid protein and FYN kinase substrate SKAP55 and a SKAP55-related protein in T cells. Proc Natl Acad Sci U S A. Jul 21 1998;95(15):8779-8784.
51. Jin X, Chen Y, Sheng Z, et al. SKAP2 is downregulated in the villous tissues of patients with missed abortion and regulates growth and migration in trophoblasts through the WAVE2-ARP2/3 signaling pathway. Placenta. 2022/10/01/ 2022;128:100-111.
52. Takahashi T, Yamashita H, Nagano Y, et al. Identification and Characterization of a Novel Pyk2/Related Adhesion Focal Tyrosine Kinase-associated Protein That Inhibits α-Synuclein Phosphorylation*. Journal of Biological Chemistry. 2003/10/24/ 2003;278(43):42225-42233.
53. Bureau JF, Cassonnet P, Grange L, et al. The SRC-family tyrosine kinase HCK shapes the landscape of SKAP2 interactome. Oncotarget. Mar 2 2018;9(17):13102-13115.
54. Mitin N, Betts L, Yohe ME, Der CJ, Sondek J, Rossman KL. Release of autoinhibition of ASEF by APC leads to CDC42 activation and tumor suppression. Nature structural & molecular biology. 2007;14(9):814-823.
55. Hamann MJ, Lubking CM, Luchini DN, Billadeau DD. Asef2 functions as a Cdc42 exchange factor and is stimulated by the release of an autoinhibitory module from a concealed C-terminal activation element. Molecular and cellular biology. 2007;27(4):1380-1393.
56. Kawasaki Y, Sagara M, Shibata Y, Shirouzu M, Yokoyama S, Akiyama T. Identification and characterization of Asef2, a guanine–nucleotide exchange factor specific for Rac1 and Cdc42. Oncogene. 2007;26(55):7620-7627.
57. Young P, Ehler E, Gautel M. Obscurin, a giant sarcomeric Rho guanine nucleotide exchange factor protein involved in sarcomere assembly. The Journal of cell biology. 2001;154(1):123-136.

58. Feng Q, Baird D, Cerione RA. Novel regulatory mechanisms for the Dbl family guanine nucleotide exchange factor Cool-2/α-Pix. The EMBO journal. 2004;23(17):3492-3504.
59. Manser E, Loo T-H, Koh C-G, et al. PAK kinases are directly coupled to the PIX family of nucleotide exchange factors. Molecular cell. 1998;1(2):183-192.
60. Mott HR, Nietlispach D, Evetts KA, Owen D. Structural analysis of the SH3 domain of β-PIX and its interaction with α-p21 activated kinase (PAK). Biochemistry. 2005;44(33):10977-10983.
61. Hoelz A, Janz JM, Lawrie SD, Corwin B, Lee A, Sakmar TP. Crystal structure of the SH3 domain of βPIX in complex with a high affinity peptide from PAK2. Journal of molecular biology. 2006;358(2):509-522.
62. Simmons A, Gangadharan B, Hodges A, et al. Nef-mediated lipid raft exclusion of UbcH7 inhibits Cbl activity in T cells to positively regulate signaling. Immunity. 2005;23(6):621-634.
63. Lim CS, Kim SH, Jung JG, Kim J-K, Song WK. Regulation of SPIN90 phosphorylation and interaction with Nck by ERK and cell adhesion. Journal of Biological Chemistry. 2003;278(52):52116-52123.
64. Ye ZS, Baltimore D. Binding of Vav to Grb2 through dimerization of Src homology 3 domains. Proc Natl Acad Sci U S A. Dec 20 1994;91(26):12629-12633.
65. Cuadrado M, Robles-Valero J. VAV Proteins as Double Agents in Cancer: Oncogenes with Tumor Suppressor Roles. Biology (Basel). Sep 8 2021;10(9).
66. Steinestel K, Brüderlein S, Steinestel J, et al. Expression of Abelson interactor 1 (Abi1) correlates with inflammation, KRAS mutation and adenomatous change during colonic carcinogenesis. PLoS One. 2012;7(7):e40671.
67. Luo K, Zhang L, Liao Y, et al. Effects and mechanisms of Eps8 on the biological behaviour of malignant tumours. Oncology Reports. 2021;45(3):824-834.
68. Smith LG, Li R. Actin polymerization: riding the wave. Current Biology. 2004;14(3):R109-R111.
69. Sekino S, Kashiwagi Y, Kanazawa H, et al. The NESH/Abi-3-based WAVE2 complex is functionally distinct from the Abi-1-based WAVE2 complex. Cell Communication and Signaling. 2015;13(1):1-15.
70. Postema MM, Grega-Larson NE, Neininger AC, Tyska MJ. IRTKS (BAIAP2L1) elongates epithelial microvilli using EPS8-dependent and independent mechanisms. Current Biology. 2018;28(18):2876-2888. e2874.
71. Li L, Baxter SS, Zhao P, Gu N, Zhan X. Differential interactions of missing in metastasis and insulin receptor tyrosine kinase substrate with RAB proteins in the endocytosis of CXCR4. Journal of Biological Chemistry. 2019;294(16):6494-6505.
72. Wu C, Cui X, Huang L, et al. IRTKS promotes insulin signaling transduction through inhibiting SHIP2 phosphatase activity. International Journal of Molecular Sciences. 2019;20(11):2834.
73. Blessing AM, Ganesan S, Rajapakshe K, et al. Identification of a Novel Coregulator, SH3YL1, That Interacts With the Androgen Receptor N-Terminus. Mol Endocrinol. Oct 2015;29(10):1426-1439.
74. Hasegawa J, Jebri I, Yamamoto H, et al. SH3YL1 cooperates with ESCRT-I in the sorting and degradation of the EGF receptor. Journal of Cell Science. 2019;132(19):jcs229179.
75. Barnett P, Bottger G, Klein AT, Tabak HF, Distel B. The peroxisomal membrane protein Pex13p shows a novel mode of SH3 interaction. The EMBO Journal. 2000;19(23):6382-6391.
76. Pires JR, Hong X, Brockmann C, et al. The ScPex13p SH3 domain exposes two distinct binding sites for Pex5p and Pex14p. Journal of molecular biology. 2003;326(5):1427-1435.
77. Kim M, Ann E, Mo J, et al. JIP1 binding to RBP-Jk mediates cross-talk between the Notch1 and JIP1-JNK signaling pathway. Cell Death & Differentiation. 2010;17(11):1728-1738.
78. Ohba T, Ishino M, Aoto H, Sasaki T. Dot far-western blot analysis of relative binding affinities of the Src homology 3 domains of Efs and its related proteins. Analytical biochemistry. 1998;262(2):185-192.
79. Deneka A, Korobeynikov V, Golemis EA. Embryonal Fyn-associated substrate (EFS) and CASS4: The lesser-known CAS protein family members. Gene. 2015;570(1):25-35.
80. Patel S, George R, Autore F, Fraternali F, Ladbury JE, Nikolova PV. Molecular interactions of ASPP1 and ASPP2 with the p53 protein family and the apoptotic promoters PUMA and Bax. Nucleic acids research. 2008;36(16):5139-5151.
81. Benyamini H, Friedler A. The ASPP interaction network: electrostatic differentiation between pro-and anti-apoptotic proteins. Journal of molecular recognition. 2011;24(2):266-274.
82. Rotem-Bamberger S, Katz C, Friedler A. Regulation of ASPP2 interaction with p53 core domain by an intramolecular autoinhibitory mechanism. PLoS One. 2013;8(3):e58470.
83. Lyraki R, Lokaj M, Soares DC, et al. Characterization of a novel RP2–OSTF1 interaction and its implication for actin remodelling. Journal of cell science. 2018;131(4):jcs211748.
84. Sottejeau Y, Bretteville A, Cantrelle F-X, et al. Tau phosphorylation regulates the interaction between BIN1's SH3 domain and Tau's proline-rich domain. Acta neuropathologica communications. 2015;3(1):1-12.
85. Lasorsa A, Malki I, Cantrelle F-X, et al. Structural basis of tau interaction with BIN1 and regulation by tau phosphorylation. Frontiers in molecular neuroscience. 2018:421.
86. Picas L, Viaud J, Schauer K, et al. BIN1/M-Amphiphysin2 induces clustering of phosphoinositides to recruit its downstream partner dynamin. Nature Communications. 2014;5(1):1-12.
87. Mendes T. Identification of the modulators of and the molecular pathways involved in the BIN1-Tau interaction, Université de Lille; 2018.
88. Yoshida Y, Kinuta M, Abe T, et al. The stimulatory action of amphiphysin on dynamin function is dependent on lipid bilayer curvature. The EMBO journal. 2004;23(17):3483-3491.

89. Chen R, Zhao H, Wu D, Zhao C, Zhao W, Zhou X. The role of SH3GL3 in myeloma cell migration/invasion, stemness and chemo-resistance. Oncotarget. 2016;7(45):73101.
90. Campiglio M, Kaplan MM, Flucher BE. STAC3 incorporation into skeletal muscle triads occurs independent of the dihydropyridine receptor. Journal of cellular physiology. 2018;233(12):9045-9051.
91. Oda Y, Otani T, Ikenouchi J, Furuse M. Tricellulin regulates junctional tension of epithelial cells at tricellular contacts through Cdc42. Journal of cell science. 2014;127(19):4201-4212.
92. Salazar MA, Kwiatkowski AV, Pellegrini L, et al. Tuba, a novel protein containing bin/amphiphysin/Rvs and Dbl homology domains, links dynamin to regulation of the actin cytoskeleton. Journal of Biological Chemistry. 2003;278(49):49031-49043.
93. Polle L, Rigano LA, Julian R, Ireton K, Schubert W-D. Structural details of human tuba recruitment by InlC of Listeria monocytogenes elucidate bacterial cell-cell spreading. Structure. 2014;22(2):304-314.
94. Cestra G, Kwiatkowski A, Salazar M, Gertler F, De Camilli P. Tuba, a GEF for CDC42, links dynamin to actin regulatory proteins. Methods in enzymology. 2005;404:537-545.
95. Kim Y-M, Stone M, Hwang TH, et al. SH3BP4 is a negative regulator of amino acid-Rag GTPase-mTORC1 signaling. Molecular cell. 2012;46(6):833-846.
96. Kim Y-M, Kim D-H. dRAGging amino acid-mTORC1 signaling by SH3BP4. Molecules and cells. 2013;35(1):1-6.
97. Acuna C, Liu X, Gonzalez A, Südhof TC. RIM-BPs mediate tight coupling of action potentials to Ca2+-triggered neurotransmitter release. Neuron. 2015;87(6):1234-1247.
98. Mencacci NE, Brockmann MM, Dai J, et al. Biallelic variants in TSPOAP1, encoding the active-zone protein RIMBP1, cause autosomal recessive dystonia. J Clin Invest. Apr 1 2021;131(7).
99. Buschman MD, Bromann PA, Cejudo-Martin P, Wen F, Pass I, Courtneidge SA. The novel adaptor protein Tks4 (SH3PXD2B) is required for functional podosome formation. Molecular biology of the cell. 2009;20(5):1302-1311.
100. de Bock CE, Hughes MR, Snyder K, et al. Protein interaction screening identifies SH 3 RF 1 as a new regulator of FAT 1 protein levels. FEBS letters. 2017;591(4):667-678.
101. Zhang P, Liu Y, Lian C, et al. SH3RF3 promotes breast cancer stem-like properties via JNK activation and PTX3 upregulation. Nature Communications. 2020/05/19 2020;11(1):2487.
102. Kärkkäinen S, van der Linden M, Renkema GH. POSH2 is a RING finger E3 ligase with Rac1 binding activity through a partial CRIB domain. FEBS Lett. Sep 24 2010;584(18):3867-3872.
103. Okur MN, Ooi J, Fong CW, et al. Intersectin 1 enhances Cbl ubiquitylation of epidermal growth factor receptor through regulation of Sprouty2-Cbl interaction. Molecular and cellular biology. 2012;32(4):817-825.
104. Hussain NK, Jenna S, Glogauer M, et al. Endocytic protein intersectin-l regulates actin assembly via Cdc42 and N-WASP. Nature cell biology. 2001;3(10):927-932.
105. Keating DJ, Chen C, Pritchard MA. Alzheimer's disease and endocytic dysfunction: clues from the Down syndrome-related proteins, DSCR1 and ITSN1. Ageing research reviews. 2006;5(4):388-401.
106. Herrero-Garcia E, O'Bryan JP. Intersectin scaffold proteins and their role in cell signaling and endocytosis. Biochimica et Biophysica Acta (BBA)-Molecular Cell Research. 2017;1864(1):23-30.
107. Tong X-K, Hussain NK, Adams AG, O'Bryan JP, McPherson PS. Intersectin can regulate the Ras/MAP kinase pathway independent of its role in endocytosis. Journal of Biological Chemistry. 2000;275(38):29894-29899.
108. Jakob B, Kochlamazashvili G, Jäpel M, et al. Intersectin 1 is a component of the Reelin pathway to regulate neuronal migration and synaptic plasticity in the hippocampus. Proceedings of the National Academy of Sciences. 2017;114(21):5533-5538.
109. Gubar O, Morderer D, Tsyba L, et al. Intersectin: the crossroad between vesicle exocytosis and endocytosis. Frontiers in endocrinology. 2013;4:109.
110. Rouka E, Simister PC, Janning M, et al. Differential recognition preferences of the three Src homology 3 (SH3) domains from the adaptor CD2-associated protein (CD2AP) and direct association with Ras and Rab interactor 3 (RIN3). Journal of Biological Chemistry. 2015;290(42):25275-25292.
111. Frese S. Structural and biochemical analysis of Nck1 and Nck2 SH2 domains2005.
112. Kesti T, Ruppelt A, Wang J-H, et al. Reciprocal regulation of SH3 and SH2 domain binding via tyrosine phosphorylation of a common site in CD3ε. The Journal of Immunology. 2007;179(2):878-885.
113. Kim TW, Kang YK, Park ZY, et al. SH3RF2 functions as an oncogene by mediating PAK4 protein stability. Carcinogenesis. 2014;35(3):624-634.
114. Lin W-H, Huang C-J, Liu M-W, et al. Cloning, mapping, and characterization of the human sorbin and SH3 domain containing 1 (SORBS1) gene: a protein associated with c-Abl during insulin signaling in the hepatoma cell line Hep3B. Genomics. 2001;74(1):12-20.
115. Matsuyama M, Mizusaki H, Shimono A, et al. A novel isoform of Vinexin, Vinexin γ, regulates Sox9 gene expression through activation of MAPK cascade in mouse fetal gonad. Genes to Cells. 2005;10(5):421-434.
116. Akamatsu M, Aota S, Suwa A, et al. Vinexin forms a signaling complex with Sos and modulates epidermal growth factor-induced c-Jun N-terminal kinase/stress-activated protein kinase activities. J Biol Chem. Dec 10 1999;274(50):35933-35937.
117. Rademacher N, Schmerl B, Lardong JA, Wahl MC, Shoichet SA. MPP2 is a postsynaptic MAGUK scaffold protein that links SynCAM1 cell adhesion molecules to core components of the postsynaptic density. Scientific Reports. 2016;6(1):1-10.

118. Krishna Subbaiah V, Massimi P, Boon SS, et al. The invasive capacity of HPV transformed cells requires the hDlg-dependent enhancement of SGEF/RhoG activity. PLoS pathogens. 2012;8(2):e1002543.
119. Zhu J, Shang Y, Zhang M. Mechanistic basis of MAGUK-organized complexes in synaptic development and signalling. Nature Reviews Neuroscience. 2016;17(4):209-223.
120. McNeil E, Capaldo CT, Macara IG. Zonula occludens-1 function in the assembly of tight junctions in Madin-Darby canine kidney epithelial cells. Molecular biology of the cell. 2006;17(4):1922-1932.
121. Cruz Garcia Y. Interactome of the β2b subunit of L-type voltage-gated calcium channels in cardiomyocytes, Universität Würzburg; 2021.
122. Quitsch A, Berhörster K, Liew CW, Richter D, Kreienkamp H-J. Postsynaptic shank antagonizes dendrite branching induced by the leucine-rich repeat protein Densin-180. Journal of Neuroscience. 2005;25(2):479-487.
123. Bucher M, Niebling S, Han Y, et al. Autism-associated SHANK3 missense point mutations impact conformational fluctuations and protein turnover at synapses. Elife. 2021;10:e66165.
124. Chakraborty S, Ain R. Nitric-oxide synthase trafficking inducer is a pleiotropic regulator of endothelial cell function and signaling. Journal of Biological Chemistry. 2017;292(16):6600-6620.
125. Zimmermann K, Opitz N, Dedio J, Renné C, Müller-Esterl W, Oess S. NOSTRIN: a protein modulating nitric oxide release and subcellular distribution of endothelial nitric oxide synthase. Proceedings of the National Academy of Sciences. 2002;99(26):17167-17172.
126. Icking A, Matt S, Opitz N, Wiesenthal A, Muller-Esterl W, Schilling K. NOSTRIN functions as a homotrimeric adaptor protein facilitating internalization of eNOS. Journal of Cell Science. 2005;118(21):5059-5069.
127. Tian L, Nelson DL, Stewart DM. Cdc42-interacting protein 4 mediates binding of the Wiskott-Aldrich syndrome protein to microtubules. Journal of Biological Chemistry. 2000;275(11):7854-7861.
128. Richnau N, Aspenström P. Rich, a rho GTPase-activating protein domain-containing protein involved in signaling by Cdc42 and Rac1. Journal of Biological Chemistry. 2001;276(37):35060-35070.
129. Quan A, Robinson PJ. Syndapin–a membrane remodelling and endocytic F-BAR protein. The FEBS journal. 2013;280(21):5198-5212.
130. Marcos T, Ruiz-Martín V, de la Puerta ML, et al. Proline-serine-threonine phosphatase interacting protein 1 inhibition of T-cell receptor signaling depends on its SH 3 domain. The FEBS journal. 2014;281(17):3844-3854.
131. Cong F, Spencer S, Côté J-F, et al. Cytoskeletal protein PSTPIP1 directs the PEST-type protein tyrosine phosphatase to the c-Abl kinase to mediate Abl dephosphorylation. Molecular cell. 2000;6(6):1413-1423.
132. Shoham NG, Centola M, Mansfield E, et al. Pyrin binds the PSTPIP1/CD2BP1 protein, defining familial Mediterranean fever and PAPA syndrome as disorders in the same pathway. Proceedings of the National Academy of Sciences. 2003;100(23):13501-13506.
133. Wu Y, Spencer SD, Lasky LA. Tyrosine phosphorylation regulates the SH3-mediated binding of the Wiskott-Aldrich syndrome protein to PSTPIP, a cytoskeletal-associated protein. Journal of Biological Chemistry. 1998;273(10):5765-5770.
134. Ren S-y, Xue F, Feng J, Skorski T. Intrinsic regulation of the interactions between the SH3 domain of p85 subunit of phosphatidylinositol-3 kinase and the protein network of BCR/ABL oncogenic tyrosine kinase. Experimental hematology. 2005;33(10):1222-1228.
135. Yudowski GA, Efendiev R, Pedemonte CH, Katz AI, Berggren P-O, Bertorello AM. Phosphoinositide-3 kinase binds to a proline-rich motif in the Na+, K+-ATPase α subunit and regulates its trafficking. Proceedings of the National Academy of Sciences. 2000;97(12):6556-6561.
136. Ito Y, Vogt PK, Hart JR. Domain analysis reveals striking functional differences between the regulatory subunits of phosphatidylinositol 3-kinase (PI3K), p85α and p85β. Oncotarget. 2017;8(34):55863.
137. Ren S-y, Bolton E, Mohi MG, Morrione A, Neel BG, Skorski T. Phosphatidylinositol 3-kinase p85α subunit-dependent interaction with BCR/ABL-related fusion tyrosine kinases: molecular mechanisms and biological consequences. Molecular and cellular biology. 2005;25(18):8001-8008.
138. Ma Y, Mi Y-J, Dai Y-K, Fu H-L, Cui D-X, Jin W-L. The inverse F-BAR domain protein srGAP2 acts through srGAP3 to modulate neuronal differentiation and neurite outgrowth of mouse neuroblastoma cells. PloS one. 2013;8(3):e57865.
139. Bacon C, Endris V, Rappold GA. The cellular function of srGAP3 and its role in neuronal morphogenesis. Mechanisms of development. 2013;130(6-8):391-395.
140. Mason FM, Heimsath EG, Higgs HN, Soderling SH. Bi-modal regulation of a formin by srGAP2. Journal of Biological Chemistry. 2011;286(8):6577-6586.
141. Newman JRB, Concannon P, Ge Y. UBASH3A Interacts with PTPN22 to Regulate IL2 Expression and Risk for Type 1 Diabetes. Int J Mol Sci. May 12 2023;24(10).
142. Feshchenko EA, Smirnova EV, Swaminathan G, et al. TULA: an SH3- and UBA-containing protein that binds to c-Cbl and ubiquitin. Oncogene. Jun 10 2004;23(27):4690-4706.
143. Tsygankov AY. TULA-family proteins: an odd couple. Cell Mol Life Sci. Sep 2009;66(17):2949-2952.
144. Kowanetz K, Crosetto N, Haglund K, Schmidt MHH, Heldin CH, Dikic I. Suppressors of T-cell receptor signaling Sts-1 and Sts-2 bind to Cbl and inhibit endocytosis of receptor tyrosine kinases. J Biol Chem. Jul 30 2004;279(31):32786-32795.
145. Janoštiak R, Tolde O, Brůhová Z, et al. Tyrosine phosphorylation within the SH3 domain regulates CAS subcellular localization, cell migration, and invasiveness. Molecular biology of the cell. 2011;22(22):4256-4267.

146. Janoštiak R, Brábek J, Auernheimer V, et al. CAS directly interacts with vinculin to control mechanosensing and focal adhesion dynamics. Cellular and molecular life sciences. 2014;71(4):727-744.
147. Gemperle J, Dibus M, Koudelková L, Rosel D, Brábek J. The interaction of p130Cas with PKN 3 promotes malignant growth. Molecular oncology. 2019;13(2):264-289.
148. Tachibana K, Urano T, Fujita H, et al. Tyrosine phosphorylation of Crk-associated substrates by focal adhesion kinase: a putative mechanism for the integrin-mediated tyrosine phosphorylation of Crk-associated substrates. Journal of Biological Chemistry. 1997;272(46):29083-29090.
149. Suzuki T, Nakamoto T, Ogawa S, et al. MICAL, a novel CasL interacting molecule, associates with vimentin. Journal of Biological Chemistry. 2002;277(17):14933-14941.
150. Tanimura S, Hashizume J, Arichika N, et al. ERK signaling promotes cell motility by inducing the localization of myosin 1E to lamellipodial tips. Journal of Cell Biology. 2016;214(4):475-489.
151. Krendel M, Osterweil EK, Mooseker MS. Myosin 1E interacts with synaptojanin-1 and dynamin via its SH3 domain. FEBS letters. 2007;581(4):644.
152. Heim JB, Squirewell EJ, Neu A, et al. Myosin-1E interacts with FAK proline-rich region 1 to induce fibronectin-type matrix. Proceedings of the National Academy of Sciences. 2017;114(15):3933-3938.
153. Matoskova B, Wong WT, Nomura N, Robbins KC, Di Fiore PP. RN-tre specifically binds to the SH3 domain of eps8 with high affinity and confers growth advantage to NIH3T3 upon carboxy-terminal truncation. Oncogene. 1996;12(12):2679-2688.
154. Matoskova B, Wong WT, Salcini AE, Pelicci PG, Di Fiore PP. Constitutive phosphorylation of eps8 in tumor cell lines: relevance to malignant transformation. Molecular and Cellular Biology. 1995;15(7):3805-3812.
155. Maa M-C, Leu T-H. EPS8, an adaptor protein acts as an oncoprotein in human cancer. Carcinogenesis: IntechOpen; 2013.
156. Jaufmann J, Franke FC, Sperlich A, et al. The emerging and diverse roles of the SLy/SASH1-protein family in health and disease—Overview of three multifunctional proteins. The FASEB Journal. 2021;35(4):e21470.
157. Kwan JJ, Slavkovic S, Piazza M, et al. HACS1 signaling adaptor protein recognizes a motif in the paired immunoglobulin receptor B cytoplasmic domain. Communications Biology. 2020/11/13 2020;3(1):672.
158. Schroeder K, Weissmann N, Brandes RP. Organizers and activators: Cytosolic Nox proteins impacting on vascular function. Free Radical Biology and Medicine. 2017;109:22-32.
159. Wishart MJ, Taylor GS, Dixon JE. Phoxy lipids: revealing PX domains as phosphoinositide binding modules. Cell. 2001;105(7):817-820.
160. Ueno N, Takeya R, Miyano K, Kikuchi H, Sumimoto H. The NADPH oxidase Nox3 constitutively produces superoxide in a p22phox-dependent manner: its regulation by oxidase organizers and activators. Journal of Biological Chemistry. 2005;280(24):23328-23339.
161. Miyano K, Ueno N, Takeya R, Sumimoto H. Direct involvement of the small GTPase Rac in activation of the superoxide-producing NADPH oxidase Nox1. Journal of Biological Chemistry. 2006;281(31):21857-21868.
162. Nakazawa S, Gotoh N, Matsumoto H, Murayama C, Suzuki T, Yamamoto T. Expression of sorting nexin 18 (SNX18) is dynamically regulated in developing spinal motor neurons. Journal of Histochemistry & Cytochemistry. 2011;59(2):202-213.
163. Park J, Kim Y, Lee S, et al. SNX18 shares a redundant role with SNX9 and modulates endocytic trafficking at the plasma membrane. Journal of cell science. 2010;123(10):1742-1750.
164. Soulet F, Yarar D, Leonard M, Schmid SL. SNX9 regulates dynamin assembly and is required for efficient clathrin-mediated endocytosis. Molecular biology of the cell. 2005;16(4):2058-2067.
165. Shin N, Lee S, Ahn N, et al. Sorting nexin 9 interacts with dynamin 1 and N-WASP and coordinates synaptic vesicle endocytosis. Journal of Biological Chemistry. 2007;282(39):28939-28950.
166. Yeow-Fong L, Lim L, Manser E. SNX9 as an adaptor for linking synaptojanin-1 to the Cdc42 effector ACK1. FEBS letters. 2005;579(22):5040-5048.
167. Baumann C, Lindholm CK, Rimoldi D, Lévy F. The E3 ubiquitin ligase Itch regulates sorting nexin 9 through an unconventional substrate recognition domain. The FEBS journal. 2010;277(13):2803-2814.
168. Schulze WX, Mann M. A novel proteomic screen for peptide-protein interactions. Journal of Biological Chemistry. 2004;279(11):10756-10764.
169. Worby CA, Simonson-Leff N, Clemens JC, Kruger RP, Muda M, Dixon JE. The sorting nexin, DSH3PX1, connects the axonal guidance receptor, Dscam, to the actin cytoskeleton. Journal of Biological Chemistry. 2001;276(45):41782-41789.
170. Lundmark R, Carlsson SR. SNX9–a prelude to vesicle release. Journal of cell science. 2009;122(1):5-11.
171. Badour K, McGavin MK, Zhang J, et al. Interaction of the Wiskott–Aldrich syndrome protein with sorting nexin 9 is required for CD28 endocytosis and cosignaling in T cells. Proceedings of the National Academy of Sciences. 2007;104(5):1593-1598.
172. Bendris N, Schmid SL. Endocytosis, Metastasis and Beyond: Multiple Facets of SNX9. Trends Cell Biol. Mar 2017;27(3):189-200.
173. Howard L, Nelson KK, Maciewicz RA, Blobel CP. Interaction of the metalloprotease disintegrins MDC9 and MDC15 with two SH3 domain-containing proteins, endophilin I and SH3PX1. Journal of Biological Chemistry. 1999;274(44):31693-31699.

174. Lupo V, Galindo MI, Martínez-Rubio D, et al. Missense mutations in the SH3TC2 protein causing Charcot-Marie-Tooth disease type 4C affect its localization in the plasma membrane and endocytic pathway. Human Molecular Genetics. 2009;18(23):4603-4614.
175. Huang C, Yi H, Zhou Y, Zhang Q, Yao X. Pan-Cancer Analysis Reveals SH3TC2 as an Oncogene for Colorectal Cancer and Promotes Tumorigenesis via the MAPK Pathway. Cancers. 2022;14(15):3735.
176. Li B, Zhuang L, Trueb B. Zyxin interacts with the SH3 domains of the cytoskeletal proteins LIM-nebulette and Lasp-1. Journal of biological chemistry. 2004;279(19):20401-20410.
177. Ma K, Wang K. Interaction of nebulin SH3 domain with titin PEVK and myopalladin: implications for the signaling and assembly role of titin and nebulin. FEBS letters. 2002;532(3):273-278.
178. Mihlan S, Reiss C, Thalheimer P, et al. Nuclear import of LASP-1 is regulated by phosphorylation and dynamic protein–protein interactions. Oncogene. 2013;32(16):2107-2113.
179. Rachlin AS, Otey CA. Identification of palladin isoforms and characterization of an isoform-specific interaction between Lasp-1 and palladin. Journal of cell science. 2006;119(6):995-1004.
180. Keicher C, Gambaryan S, Schulze E, Marcus K, Meyer HE, Butt E. Phosphorylation of mouse LASP-1 on threonine 156 by cAMP-and cGMP-dependent protein kinase. Biochemical and biophysical research communications. 2004;324(1):308-316.
181. Orth MF, Cazes A, Butt E, Grunewald TG. An update on the LIM and SH3 domain protein 1 (LASP1): a versatile structural, signaling, and biomarker protein. Oncotarget. 2015;6(1):26.
182. Butt E, Raman D. New frontiers for the cytoskeletal protein LASP1. Frontiers in oncology. 2018;8:391.
183. Esmailzadeh S, Jiang X. AHI-1: a novel signaling protein and potential therapeutic target in human leukemia and brain disorders. Oncotarget. 2011;2(12):918.
184. Hou W, Nemitz S, Schopper S, Nielsen ML, Kessels MM, Qualmann B. Arginine methylation by PRMT2 controls the functions of the actin nucleator Cobl. Developmental Cell. 2018;45(2):262-275. e268.
185. Imbastari F, Dahlmann M, Sporbert A, et al. MACC1 regulates clathrin-mediated endocytosis and receptor recycling of transferrin receptor and EGFR in colorectal cancer. Cellular and Molecular Life Sciences. 2021;78(7):3525-3542.
186. Stein U, Walther W, Arlt F, et al. MACC1, a newly identified key regulator of HGF-MET signaling, predicts colon cancer metastasis. Nature medicine. 2009;15(1):59-67.
187. Kim H, Oh H, Oh YS, et al. SPIN90, an adaptor protein, alters the proximity between Rab5 and Gapex5 and facilitates Rab5 activation during EGF endocytosis. Experimental & Molecular Medicine. 2019/07/01 2019;51(7):1-14.
188. Ackermann A, Brieger A. The role of nonerythroid spectrin II in cancer. Journal of oncology. 2019;2019.
189. Guo X, Chen SY. Dedicator of cytokinesis 2 in cell signaling regulation and disease development. Journal of cellular physiology. 2017;232(8):1931-1940.
190. Sakurai T, Kukimoto-Niino M, Kunimura K, et al. A conserved PI (4, 5) P2–binding domain is critical for immune regulatory function of DOCK8. Life science alliance. 2021;4(4).
191. Huang M, Liang C, Li S, et al. Two autism/dyslexia linked variations of DOCK4 disrupt the gene function on rac1/rap1 activation, neurite outgrowth, and synapse development. Frontiers in cellular neuroscience. 2020:577.
192. Lei Y, Xu J, Li M, et al. MIA SH3 Domain ER Export Factor 3 Deficiency Prevents Neointimal Formation by Restoring BAT-Like PVAT and Decreasing VSMC Proliferation and Migration. Frontiers in Endocrinology. 2021;12.
193. Wodarczyk C, Distefano G, Rowe I, et al. Nephrocystin-1 forms a complex with polycystin-1 via a polyproline motif/SH3 domain interaction and regulates the apoptotic response in mammals. PloS one. 2010;5(9):e12719.
194. Mannella V, Quilici G, Nigro EA, et al. The N-Terminal Domain of NPHP1 Folds into a Monomeric Left-Handed Antiparallel Three-Stranded Coiled Coil with Anti-apoptotic Function. ACS Chemical Biology. 2019;14(8):1845-1854.
195. Benzing T, Simons M, Walz G. Wnt signaling in polycystic kidney disease. Journal of the American Society of Nephrology. 2007;18(5):1389-1398.
